# *ALK* rearrangement: a high-frequency alteration in ovarian metastasis from lung adenocarcinoma

**DOI:** 10.1186/s13000-019-0864-7

**Published:** 2019-08-28

**Authors:** Rui Bi, Qianming Bai, Xiaoli Zhu, Xiaoyu Tu, Xu Cai, Wenhua Jiang, Xiaoli Xu, Shaoxian Tang, Huijuan Ge, Bin Chang, Yufan Cheng, Hualei Gan, Xiaoyan Zhou, Wentao Yang

**Affiliations:** 1 Department of Pathology, Fudan University Shanghai Cancer Center, Fudan University, 270 Dong An Road, Shanghai, 200032 China; 2 Department of Oncology, Shanghai Medical College, Fudan University, 270 Dong An Road, Shanghai, 200032 China

**Keywords:** Ovary, Metastatic lung adenocarcinoma, *ALK* rearrangement

## Abstract

**Background:**

Ovarian metastatic tumors from lung adenocarcinoma are rare, and a serial study of these tumors is lacking to date. Additionally, a better understanding of the clinicopathological and molecular characteristics of metastatic tumors is needed.

**Methods:**

Seven cases of ovarian metastasis from lung adenocarcinoma from 2013 to 2017 at our institute were investigated. The results were combined with those found in literature review. A total of 16 cases were analyzed in the present study. We examined clinicopathological and immunohistochemical characteristics, further detected *ALK* rearrangement by FISH (fluorescence in situ hybridization), and assessed *EGFR* and *KRAS* mutations using Sanger sequencing or the amplification refractory mutation system (ARMS).

**Results:**

The mean age of the patients was 44.6 years (range, 33–56 years). Eleven of sixteen patients developed ovarian tumors within a mean time of 18.5 months (range, 5–48 months) from the initial diagnosis of lung adenocarcinoma; 5 patients had lung tumors and ovarian masses simultaneously. Five tumors (5/16, 31%) occurred in the bilateral ovaries, and the others were unilateral ovarian tumors (11/16, 69%). All seven cases from our institute were positive for TTF-1 and Napsin A but negative for PAX8. In four cases, ALK (D5F3) was diffusely and strongly expressed, with *ALK* rearrangements (4/7, 57%). Overall, *ALK* rearrangement was found by FISH or immunohistochemistry in 11/16 (69%) cases. In two cases, *EGFR* mutations in exons 19 and 21, respectively, were found. One patient did not detected *EGFR* or *ALK* mutation in the metastatic tumor, but the primary lung adenocarcinoma did harbor an *EGFR* mutation. Two cases had no alterations in three genes above. Although the mean survival time of the patients with ALK rearrangement was longer than those without (mean survival time 25 m vs. 20 m), no statistical significance of the difference was found.

**Conclusions:**

As the largest case series of ovarian metastasis from lung adenocarcinoma, our findings indicate that *ALK* rearrangement is the most common molecular alteration. Although patients with *ALK* rearrangement appear to have a better prognosis than do those without *ALK* rearrangement, more cases with longer follow-up and multivariant analysis are needed to clarify this point.

## Background

Lung cancer is the second most common cancer, and most cases are diagnosed at advanced stages. The 5-year survival rate of lung cancer is less than 20%. Lung cancer in non-smokers appears to be a distinct disease caused by driver mutations that are different from the genetic changes observed in lung cancer in smokers. In western countries, rearrangements in the gene (*ALK*) encoding anaplastic lymphoma receptor tyrosine kinase have been found in only 3–7% of lung adenocarcinomas; in contrast, *EGFR* mutation is approximately 10–15%, more frequent than *ALK* rearrangement [1, 2]. Nevertheless, these two alterations appear to be mutually exclusive in patients [3, 4], and only recent rare cases have shown concomitant alterations in *ALK* and *EGFR* [5, 6].

Ovarian metastatic tumors derive from many sites. The common primary sites include gastrointestinal tract, pancreas, and gynecologic tract (the cervix and uterus) [7]. However, lung carcinoma metastasis to the ovary is rarely reported. Reports of these metastatic tumors have included all major lung cancer histotypes, with small cell carcinomas comprising the largest proportion, followed by adenocarcinomas, large cell carcinomas and squamous cell carcinomas [8]. As stated above, lung adenocarcinoma metastases to the ovary are rare, and the incidence is not yet clear. Lung metastasis to uncommon sites occurs in less than 5% of cases, with metastatic adenocarcinoma to the ovary occurring in only 0.07%. So the frequency of total lung tumor metastasis to the ovary is estimated to be less than 0.01% [9]. More remarkably, although ovarian metastasis from lung carcinoma has an extremely low incidence, *ALK* rearrangement in these patients has occasionally been reported [10–17].

In the present study, we investigated 7 consecutive cases of lung adenocarcinoma metastasis to the ovary at our institute with cases-review in the literature. We obtained clinicopathological data regarding major molecular alterations for targeted therapy. To the best of our knowledge, this study is the largest panel of ovarian metastatic tumors from lung adenocarcinomas focusing on genetic alterations to date.

## Methods

### Samples

Seven cases of lung carcinoma metastasis to the ovary, including 5 treated with surgery at Fudan Cancer Center and 2 consultation cases, were reviewed in the Department of Pathology of Fudan University Shanghai Cancer Center in 2013–2017. Clinical information and gross features were obtained from medical records. Formalin-fixed, paraffin-embedded tissue blocks or unstained slides were reprocessed for hematoxylin and eosin staining, immunohistochemistry and molecular analysis. All cases were reviewed by two senior pathologists who verified the diagnosis. The present study was approved by our institutional ethics committee. We also reviewed unselected reported cases of ovarian metastasis from lung adenocarcinoma in the English literature in PubMed, and 9 cases with molecular alterations were retrieved. In total, 16 cases were analyzed in the present study.

### Immunohistochemistry

Immunohistochemistry analyses, including detection of PAX8, ER, PR, TTF-1, Napsin A, ALK (D5F3), CK7, CK20, CDX2 and HNF-1β, were performed for all 7 cases using a Ventana Benchmark XT autostainer (Ventana Medical Systems Inc., Tucson, AZ, USA). Appropriate positive and negative controls were included. A list of antibodies used in this study is shown in Table 1.

**Table 1:** Antibodies used for immunohistochemistry

Antibody	Dilution	Clone	Manufacturer
TTF-1	Ready-use	SPT24	Leica
NapsinA	1:500	KCG1.1	Abcam
ALK	Ready-use	D5F3	Ventana
PAX8	1:150	MRQ-50	Cell Marque
ER	Ready-use	SP1	Ventana
PR	Ready-use	1E2	Ventana
CK7	1:100	SP52	Ventana
CK20	Ready-use	SP33	Ventana
CDX2	1:50	EPR2764Y	Maxim
HNF-1β	1:500	polyclone	Sigma
P63	Ready-use	4A4	Roche
P40	Ready-use	ER8	Maxim

### *EGFR* and *KRAS* mutational analysis

The mutational statuses of *EGFR* (exons 18, 19, 20 and 21) and *KRAS* (exon 2) were determined by polymerase chain reaction (PCR)-based direct gene sequencing, as previously described [18], or by the amplification refractory mutation system (ARMS). In brief, genomic DNA from the 7 tumors was extracted using a QIAamp DNA FFPE Tissue Kit (Qiagen, Valencia, CA, USA) according to the manufacturer’s instructions. The primers used for *EGFR* and *KRAS* are listed in Table 2. The PCR products were confirmed by agarose gel electrophoresis, purified using a DNA Clean/Extraction Kit (GeneMarkBio, A&D Technology, Beijing, China), and submitted for direct sequencing using a BigDye Terminator Cycle Sequencing Kit (Applied Biosystems) according to the manufacturer’s protocol. The sequencing products were ethanol precipitated before being assessed using a 3500 Genetic Analyzer (Applied Biosystems), and the resulting sequence data were analyzed using Chromas software. Each mutation was verified in both the sense and antisense directions and was independently evaluated by two investigators. ARMS was carried out according to the manufacturer’s instructions (AmoyDx of Xiamen, Fujian Province, China).

**Table 2:** The primers of *EGFR* and *KRAS* on hot spots

*EGFR*	
Exon 18	F: 5′-AGCATGGTGAGGGCTGAGGTGAC-3’
	R: 5′-ATATACAGCTTGCAAGGACTCTGG-3’
Exon 19	F: 5′-CCAGATCACTGGGCAGCATGTGGCACC-3’
	R: 5′-AGCAGGGTCTAGAGCAGAGCAGCTGCC-3
Exon 20	F: 5′-GATCGCATTCATGCGTCTTCACC-3’
	R: 5′- TTGCTATCCCAGGAGCGCAGACC-3
Exon 21	F: 5′-TCAGAGCCTGGCATGAACATGACCCTG-3’
	R: 5′- GGTCCCTGGTGTCAGGAAAATGCTGG-3’
*KRAS*	
Exon 2	F: 5′- AGGCCTGCTGAAAATGACTG-3’
	R: 5′-TCAAAGAATGGTCCTGCACC-3’

### *ALK* rearrangement based on FISH

Vysis *ALK* Break Apart FISH Probe Kit (Abbott Molecular) was used for *ALK* testing, as described previously [19]. Fifty or more non-overlapping nuclei were counted by two professional molecular pathologists according to standard criteria in our laboratory. The positive cut-off for *ALK* rearrangement was at least 15% tumor cells with a split pattern and/or single orange signal without a corresponding green signal [20]. An increased number of nuclei or alterative nuclei were counted if the results were close to the cut-off values.

## Results

### Clinicopathological data

Among the seven cases in our cohort and the nine cases previously reported, patient ages ranged from 33 to 56 years (mean 44.6 years). Among 12 patients for which smoking history was known, 1 patient was a light smoker (2.5-pack years), 1 patient smoked 1-pack per day, and the other patients had no smoking history (10/12, 83%). In 5 cases (5/16, 31%), the lung adenocarcinoma and ovarian metastatic tumors occurred simultaneously. For the remaining 11 cases (11/16, 69%), ovarian tumors were observed at 5–48 months (mean 18.5 months) following the diagnosis of the lung adenocarcinoma. Computed tomography revealed bilateral ovarian tumors in only five patients (5/16, 31%); the others presented unilateral tumors (11/16, 69%). Concurrent metastatic sites were present in most cases (13/16, 81%), including the bone (6 cases), brain (5 cases), supraclavicular lymph node (3 cases), liver (2 cases), and pleural lymph node (1 case) but were absent in 3 cases (3/16, 19%) (Table 3). Serum CA125 was elevated with median values in the range of 100–500 U/ml in 5 recorded cases.

**Table 3:** Clinicopathological features of seven ovarian metastatic lung adenocarcinoma

Case	Age (years)	Smoking history	Side	Tumor size (cm)	Surgery	Gross appearance	Morphological features	Interval time between primary lung cancer and matastasis to ovary and concurrent metastatic sites	ALK (D5F3)	ALK-FISH	EGFRstatus	KRAS status	Targeted treatment	Follow-up
LTO_1	43	N/A	U	L,11*7*3	TAH-BSO	solid, with minor honeycomb cut-section	solid	30 m, no concurrent sites	Positive	Rearrangement	Neg.	Neg.	No	10 m, DOD
LTO_2	39	no	B	L,4*4*3;R,4*3*2	TAH-BSO and omentectomy	solid	acinar	5 m, no concurrent sites	negative	No	exon 19 mutation	Neg.	Gefitinib	13 m, DOD
LTO_3	52	no	U	L, 21.5*18*8	TAH-BSO	Cystic prominent, focal solid area	Acinar+solid	Synchronous, bone, left supraclavicular lymph node	negative	No	Neg.	Neg.	No	36 m, alive
LTO_4	50	N/A	U	L,16*15*6	USO	solid and cystic section, focal papillary architeched	acinar	6 m, brain and bone	Positive	Rearrangement	Neg.	Neg.	No	19 m, alive
LTO_5	56	no	B	R, 8.5*5*4.5;L, 3*2*1	BSO + appendectomy	solid,	acinar	19 m, bone	Positive	Rearrangement	Neg.	Neg.	No	22 m, alive
LTO_6	35	no	U	/	TAH-BSO	/	solid predominant	Synchronous, brain and bone	Positive	Rearrangement	Neg.	Neg.	Crizotinib	24 m, alive
LTO_7	55	no	U	R, 14*10*9	/	Cystic with old dark brown viscous liguid	acinar	7 m, liver	negative	No	Neg.	Neg.	No	8 m, DOD
LTO_8^(10)^	54	2.5-pack year	B	R, 2–4.1 L, 2.7–5	BSO	/	Thick trabeculae or solid nests	1.2 m, brain	Positive	Rearrangement	Neg.	Neg.	No	/
LTO_9^[11]^	39	1-pack per day	B	15 by 10	Left salpingo-oophorectomy+right ovarian cystectomy	Half cystic and half solid, filled with old blood in cyst and slightly yellow and white solid tissue with small cysts	acinar	20 m, brain	/	Rearrangement	/	/	/	28 m, alive
LTO_10^[12]^	50	/	B	12.8*12.2*11.7	TAH-BSO	Mixed cystic and solid adnexal mass	Solid with Intracytoplasmic mucinous and signet-ring cells	39 m, liver and bone	/	Rearrangement	Neg.	/	Crizotinib	/
LTO_11^[13]^	38	No	U	L	/	/	/	Synchronous, bone	/	Neg.	exon 21 mutation		Erlotinib	24 m, DOD
LTO_12^[13]^	47	/	U	R	BSO	solid	solid predominant	20 m, right supraclavicular lymph node	/	Rearrangement	Neg.	/	Crizotinib	12 m, alive
LTO_13^[14]^	33	No	U	L:7.9*6.9	Adnexal mass biopsy	solid	acinar	Synchronous, bilateral supraclavicular lymph node	Positive	/	Neg.	/	Crizotinib	4 m, alive
LTO_14^[15]^	41	No	U	L: 10	Left salpingo-oophorectomy	solid	acinar	8 m, pleural	/	Rearrangement	Neg.	Neg.	Crizotinib	10 m, alive
LTO_15^[16]^	45	No	U	R:11	TAH-BSO	Solid	Solid nests with abundant granular and extensive signet ring cell change	48 m, no concurrent sites	/	/	Mutation in lung cancer	/	/	1 m, DOD
LTO_16^[17]^	37	No	U	L: 9*5.8	No resection	Mixed solid and cysts	Acinar and several signet-ring cells	Synchronous, brain	Pos. in lung cancer	Rearrangment in lung cancer	/	/	Alectinib	20 m, alive

Abbreviation: *TAH-BSO* total abdominal hysterectomy and bilateral salpingo-oophorectomy, *m* month, *N/A* not applicable, *U* unilateral, *B* bilateral, *DOD* dead of disease, *L* left, *R* Right

### Pathological features

**Fig. 1:**
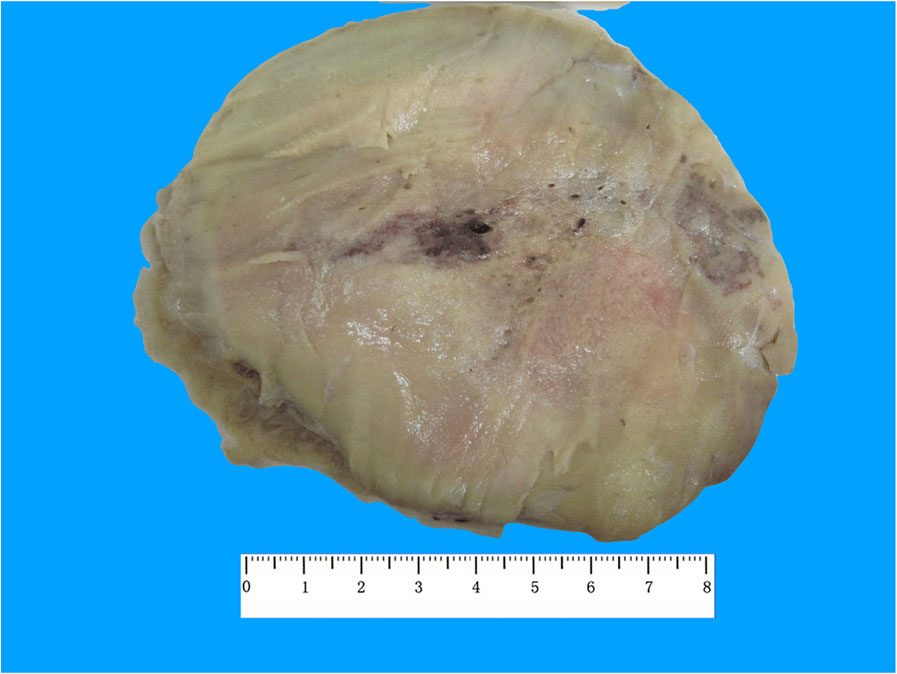
LTO_1 showed a solid tumor with a grey-yellow cut surface and focal hemorrhage

On gross examination, the ovarian tumors exhibited smooth outer surface. Macroscopic omental cake was present in one case (LTO_2). The mean size of 13 ovarian tumors with available data was 11 cm (range, 4–21.5 cm). The cut surface was predominantly solid in 7 cases, whereas it was cystic in two cases; the other 4 cases included mixed solid and cystic surfaces. The solid area of the tumor had a medium firmness level (LTO_1, Fig. 1). Large cavities in two of the tumors (LTO_3 and 7) were filled with dark brown viscous liquid and showed partial papillary architecture. The clinical and macroscopic features of all cases are summarized in Table 3.

**Fig. 2:**
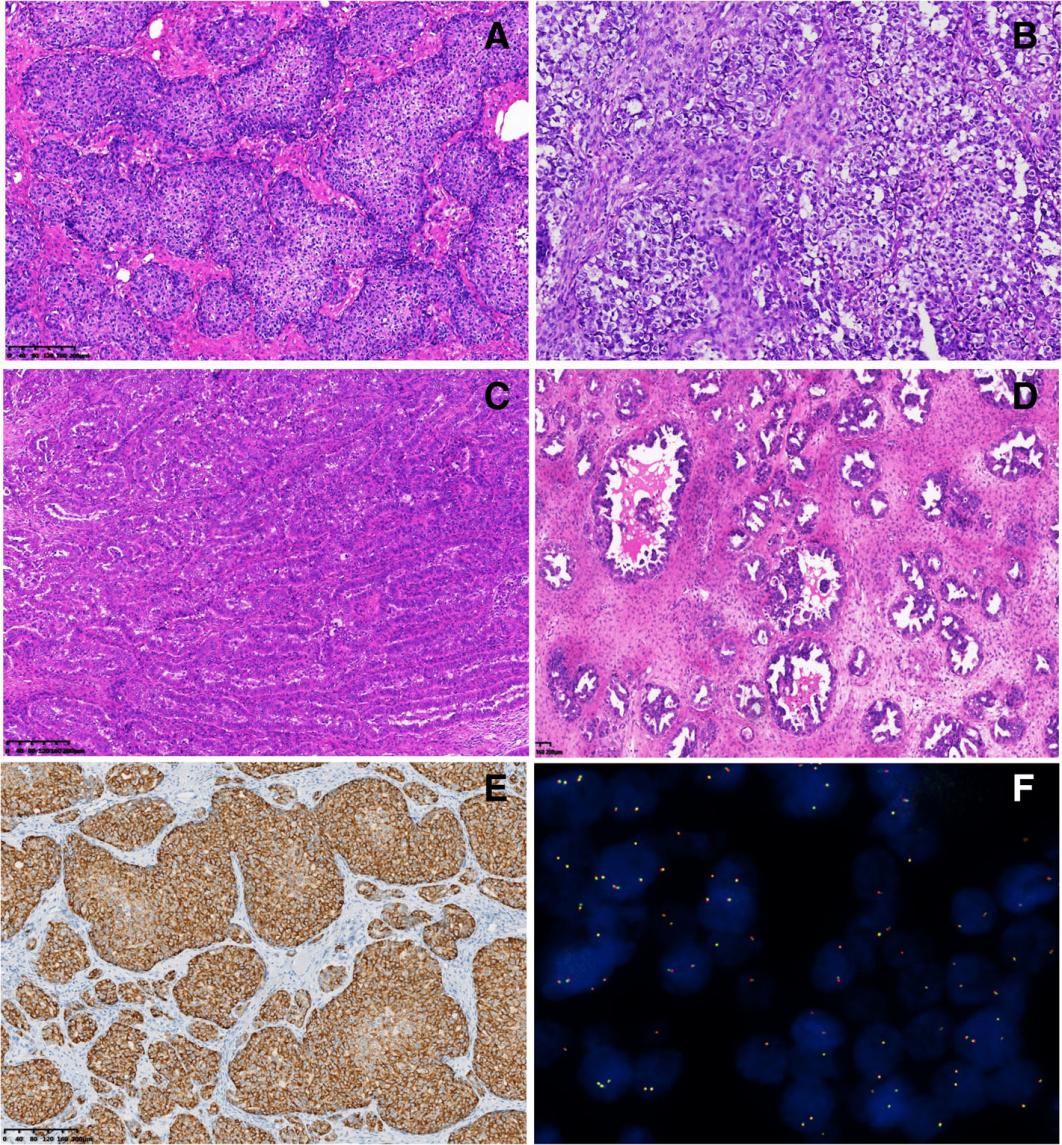
Histological subtypes of ovarian metastatic lung adenocarcinomas. **a** and **b**, LTO_1 and LTO_6, solid subtype; **c** and **d**, LTO_4 and LTO_5, acinar subtype; **e**. ALK (D5F3) diffuse expression in LTO_1; **f**. ALK rearrangement with split orange and green signals by FISH

Upon microscopic observation, all 16 cases were found to be adenocarcinomas, including 5 cases with solid or prominent solid features, 8 cases with prominent acinar features (Fig. 2a-d) and 2 cases with a mixed solid and acinar pattern. One case did not represented the histological subtype in the article. LTO_1 was characterized by solid features, and the tumor cells had distinct cell membrane and little mucin, similar to squamous cell carcinoma, but were negative for p63 and p40. Prominent solid patterns and sheets of tumor cells were also observed in other four cases, presenting polymorphic epithelioid cells with eosinophilic or clear cytoplasm and unclear cell borders. Remarkably, signet ring cells were observed in 3 cases. Tumor cells with an eosinophilic cytoplasm were attached to the cystic wall and exhibited hemorrhage and inflammatory infiltration, with a mixed acinar and solid growth pattern in LTO_3. The acinar features included long tubules but with little mucus in the lumen in 8 cases. Signet ring cells were observed in LTO_16. Additionally, acinar features with micropapillaries in acinar cells were found in LTO_5, and eosinophilic, mucinous fluid was observed in some acinar cells. In LTO_7, prominent mucin were present in acinar cells, which showed a cribriform pattern. In the present case series, the acinar pattern was slightly more common than solid pattern. However, 4/5 cases with solid features harbored *ALK* rearrangement.

### Immunohistochemistry

ER, PR, HNF-1β and PAX8 expression was negative in our 7 cases, while TTF-1, Napsin A and CK7 were expressed in all 7 cases. No cases were positive for CK20 or CDX2. LTO_1 was negative for p63 and p40. Four of 7 cases showed diffuse and strong positivity for ALK (D5F3) (Fig. 2e). Interestingly, the 2 cases with solid or prominent solid features were both ALK positive. The immunohistochemistry results are presented in Table 3.

### Genetic alterations in *EGFR* and *KRAS* and *ALK* rearrangement

**Fig. 3:**
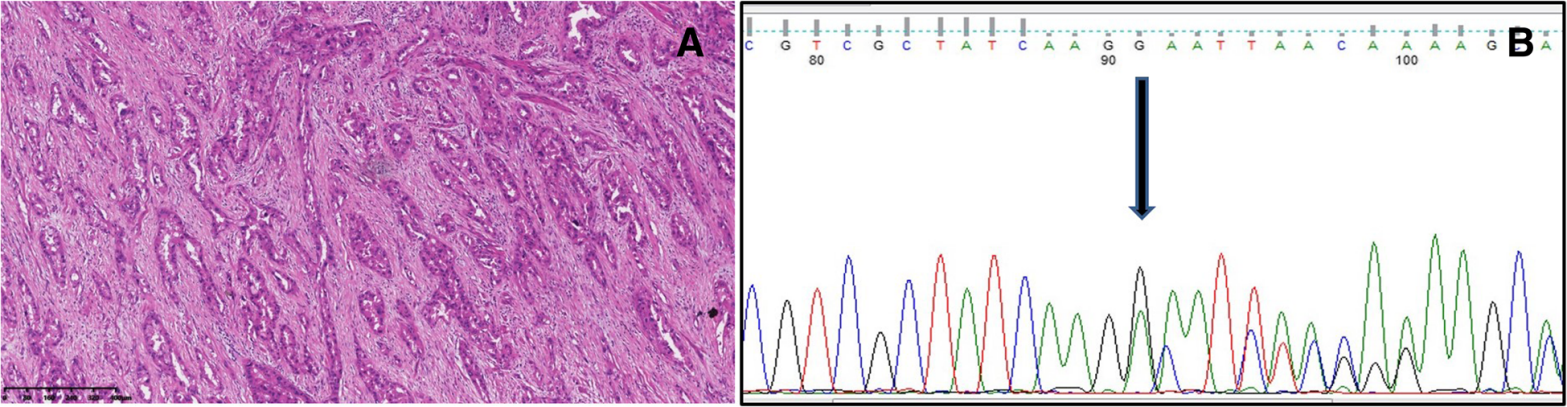
**a**. Case LTO_2 showed an acinar subtype. **b**. EGFR mutation in exon 19, p.746-750del

In seven cases, sequencing was successful and revealed mutations in *EGFR* exons 18, 19, 20 and 21 and *KRAS* exon 2; a frameshift mutation of *EGFR* exon 19 was detected in one of seven cases (Fig. 3, Case LTO_2). Wild-type *EGFR* and *KRAS* genes were found in the remaining 6 cases, though *ALK* rearrangement was present in 4. Among all 16 cases, FISH or immunohistochemistry revealed an *ALK* rearrangement in 10. For one patient (LTO_16), molecular examination of the ovarian tumor was not performed, but the lung cancer harbored an *ALK* rearrangement. Based on the high concordance of *ALK* status between the primary and metastatic tumors [20], we presumed A*LK* rearrangement in 11/16 (68.8%) cases in the present study. Two cases showed *EGFR* mutation in exon 19 and 21, respectively. One patient did not exhibit an *EGFR* mutation or *ALK* rearrangement in the metastatic tumor, but the primary lung adenocarcinoma did carry an *EGFR* mutation. The remaining 2 cases had no *EGFR* or *KRAS* mutation or *ALK* rearrangement.

### Follow-up

**Fig. 4:**
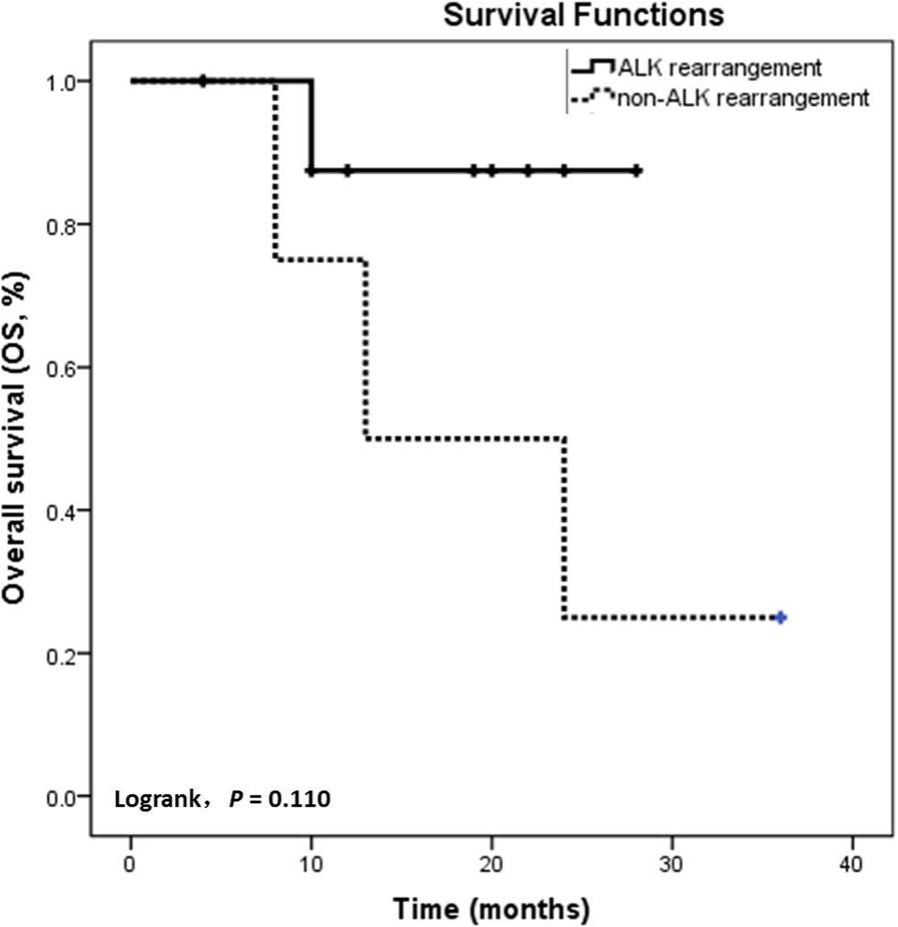
Kaplan-Meier survival analysis and log-rank analysis of patients with or without *ALK* rearrangements. Patients with *ALK* rearrangement had a better prognosis than did those without *ALK* rearrangement, though no statistically significant difference in survival (log-rank test, *P* = 0.110) was observed due to the small cohort. One patient who did not undergo targeted therapy for *ALK* rearrangement died. To some extent, *ALK* inhibitors are effective for these patients

The 7 patients in our cohort were followed up until June 7, 2019. The follow-up time of the 9 patients from the English literature were obtained from the original articles. All 16 patients underwent chemotherapy, and 8 received targeted drugs: gefitinib and Tarceva for *EGFR* mutations and crizotinib and alectinib for *ALK* rearrangements. Five patients died in 10 months (m), 13 m, 8 m, 24 m and 1 m, after the diagnosis of ovarian metastasis from lung adenocarcinoma, including a patient harboring an *ALK* rearrangement (LTO_1) who did not undergo targeted therapy and had hypothyroidism for 3 years. Two patients, both carrying an *EGFR* mutation (LTO_2 and LTO_11), were treated with gefitinib or Tarceva. One patient (LTO_15) exhibited an *EGFR* mutation in the lung tumor but the mutation status of the ovarian metastatic tumor was unknown. The fifth patient who died (LTO_7) exhibited no *ALK/EGFR* alterations and did not undergo targeted therapy. Of the 9 surviving patients, 8 had *ALK* rearrangements. Hence, two groups were formed based on the molecular alterations present. The first group included 11 patients with *ALK* rearrangements. The mean survival of this group was 25 m, with only 1 death from the disease. The other group of 5 patients did not have *ALK* rearrangements; the mean survival was 20 m, but only 1 patient survived. Although survival was not significantly different between the two groups (*P* = 0.110), longer follow-up with additional samples may clarify the difference in survival between these two groups (Fig. 4). Additional details are listed in Table 3.

## Discussion

The most common non-small cell lung cancer (NSCLC) metastatic sites are the brain [21], followed by the bone and liver [22]. All other organ metastases comprise less than 5% of cases, and thus, they may be defined as uncommon metastases. The anatomical sites in decreasing order of frequency are the soft tissue, kidney, pancreas, spleen, peritoneum, intestine, bone marrow, eye, ovary, thyroid, heart, breast, tonsil nasal cavity [9], and gastric region [23]. The total frequency of lung metastasis to the ovary is estimated to be less than 0.01% [9]. Due to the paucity of reported cases, the clinicopathological features of these tumors remain unknown. To the best of our knowledge, this is the largest number of cases of ovarian metastasis from lung adenocarcinoma examined to date. Although the ovary is an uncommon metastatic site in the female reproductive tract, uterine cervix metastasis from lung adenocarcinoma harboring *ALK* rearrangement has been reported [24], as have concurrent cervix and breast metastases [25]. In our cohort, the minority (19%) of ovarian metastatic tumors involved only one metastatic site, whereas the majority of ovarian metastatic tumors were concurrent with other metastatic sites (81%), especially the bone and brain.

Ovarian mucinous carcinoma accounts for only 3–4% of all primary tumors [26], and most cases constitute metastatic tumors from diverse sites. As previously noted by Lee et al. [27], metastatic tumors of the ovary tend to show bilateral masses. However, unilateral masses were common in our cohort of metastatic lung adenocarcinomas (11/16, 68.8%), in contrast to Krukenberg tumors with bilateral masses. This is consistent with unilateral ovarian metastasis in twelve of nineteen cases in Irving’s report [8]. Furthermore, tumors greater than 10 cm in size tended to be primary ovarian tumors, but the mean size in the present study was 11 cm. These findings indicate that ovarian metastatic tumors from lung adenocarcinomas have distinct clinicopathological features compared with those from other organs, and some characteristics may result in misdiagnosis of primary ovarian tumors.

Metastatic mucinous carcinomas mainly include those of the gastrointestinal tract, pancreatic, and gynecologic organs [27, 28]. Lung adenocarcinoma metastases to the ovary are extremely rare and comprise less than 0.01% of cases [9]. Although metastatic ovarian tumors prior to the primary lung adenocarcinomas were not found in our cohort, 5 cases (16%) of ovarian tumors were reported prior to the lung carcinoma, including 1 adenocarcinoma [8]. Hence, differentiating primary ovarian tumors from metastatic tumors is a major challenge, especially for mucinous ovarian carcinomas. A panel of immunohistochemical markers should be used for differential diagnosis. PAX8 is recognized as a sensitive marker of the gynecologic tract [29], but it was absent in all 7 cases in our cohort. TTF-1 and Napsin A, which are commonly-used pulmonary-origin markers, are helpful in distinguishing these cases from cases of primary ovarian mucinous carcinoma [30]. TTF-1 and Napsin A were expressed in all 7 cases, and metastatic lung adenocarcinoma was further supported with the negative results for PAX8, HNF-1β, ER and PR, and history of lung adenocarcinoma.

Dominant oncogenes in NSCLC are associated with different biological behaviors manifesting as distinct patterns of metastasis, and *ALK* rearrangement predisposes to rare sites of pericardial and pleural disease [31, 32]. Interestingly, in the present study, 11/16 (69%) cases harbored *ALK* rearrangements in an unselected rare ovarian metastasis from a lung adenocarcinoma as the most frequent molecular alteration. *EML4-ALK* fusion genes are predominantly observed in younger, nonsmoking/light smoking female populations, especially in East Asia. Histopathologically, this *ALK* rearrangement in lung adenocarcinoma appears to promote the unique features of solid and/or signet ring cells [33]. Other features have been found in cases with *ALK* rearrangements, whereby mixed subtype adenocarcinomas were the most common subtype, followed by the acinar predominant subtype, papillary predominant subtype [34], micropapillary and cribriform growth [35, 36], bronchioloalveolar carcinoma (BAC) and low-grade endobronchial mucoepidermoid carcinoma. Rare casesof adenosquamous carcinoma and mucoepidermoid carcinoma has been reported [35]. Although there is one reported case of ovarian metastatic lung adenocarcinoma with comprehensive solid and signet ring cells [16], regrettably, there was no further genetic analysis of this case. Our results also indicated that the solid phenotype is highly suggestive of *ALK* alteration, and 4/5 cases with solid features harbored *ALK* rearrangement. However, the acinar pattern was slightly more common than was the solid pattern in patients with *ALK* rearrangement (6 acinar vs. 5 solid), which is consistent with previous reports in which lung adenocarcinoma with *ALK* rearrangement was likely to be related to acinar components [37].

Notably, lung cancer with *ALK* rearrangement represents only a small subset of NSCLC, but metastatic ovarian tumors were found to occur in most *ALK* fusion-positive cases. Why the ovary is the favored site of these lung cancers is not known. In our cohort, the ALK protein was expressed in 4 cases (57%), and 100% concordance with the FISH results was observed. Most evidence demonstrates good correlation between the immunohistochemical staining of ALK (clone D5F3) and FISH [38]. To reduce the economic burden, patients should be screened by *ALK* immunohistochemistry to ensure whether they are suitable for targeted therapy. Notably, pleural metastatic tumors weakly express *ALK* (clone D5F3), but 75% were positive for ALK rearrangement by FISH; therefore, the results of immunohistochemical and FISH may be inconsistent for distant metastases [38].

Recently, Gupta R et al. demonstrated a decreased median survival rate for *ALK*-positive tumors with uncommon sites of metastasis compared with common sites [31]. However, only one patient with ovarian metastatic *ALK*-positive tumors was reported in their paper (this was not reanalyzed in the current study due to lack of detail information). According to our data, slightly increased survival compared with triple-negative (no EGRF/KRAS/ALK alterations) and EGFR/KRAS mutation cases may be associated with uncommon metastatic ovary tumors with ALK rearrangement, though this difference was not statistically significant. As mentioned, patients with *ALK* rearrangement have a better prognosis than do those with wild-type *EGFR* [39]. Hence, patients with *ALK* rearrangement and uncommon sites of metastasis to the ovary may achieve better outcomes and longer survival times. Because of the paucity of ovarian metastasis from lung adenocarcinoma, longer follow-up times and more samples are needed for further research.

The *EGFR* mutation is approximately 10–15%, more frequent than *ALK* rearrangement [1, 2]. Histologically, patients with *EGFR* mutation more frequently display BAC or papillary components, but the incidence of solid features is lower than that in other patients [40, 41]. One patient in our group with an *EGFR* mutation in exon 19 (p.746-750del) showed the acinar subtype, which conformed to the morphological features, and the mutation was in concordance with that of the primary lung adenocarcinoma. According to previous reports, there is an approximate discordance rate of 16.2–27% between primary lung adenocarcinomas and corresponding metastases. One case showed L858R in a primary lung cancer but 2235-2249del in ovarian metastatic tumors [42]. Because the responsiveness to *EGFR* tyrosine kinase inhibitors tends to correlate with the *EGFR* mutation status in metastatic lesions compared with primary tumors [42, 43], metastatic tumors should be retested. Patients with rare *EGFR* mutations in metastatic ovarian carcinomas are likely to have a worse prognosis than are those with tumors with *ALK* rearrangement.

## Conclusions

In summary, our findings indicate that *ALK* rearrangement is the most common molecular alteration in lung tumor metastasis to the ovary, followed by *EGFR* mutation. Although patients with ALK rearrangement appear to have a better prognosis than do those without ALK rearrangement, more cases with longer follow-up and multivariant analysis are needed to clarify this point.

## Data Availability

All data generated or analyzed during this study are included in this published article.

## Abbreviations

ALK: anaplastic lymphoma receptor tyrosine kinase
ARMS: amplification refractory mutation system
BAC: bronchioloalveolar carcinoma
NSCLC: Non-small-cell lung cancer
